# Viscoelastic bandgap in multilayers of inorganic–organic nanolayer interfaces

**DOI:** 10.1038/s41598-022-14257-z

**Published:** 2022-06-24

**Authors:** Rajan Khadka, Ganpati Ramanath, Pawel Keblinski

**Affiliations:** Department of Materials Science and Engineering, Rensselaer Polytechnic Institute, Troy, NY 12180 USA

**Keywords:** Organic-inorganic nanostructures, Structural properties

## Abstract

Incorporating molecular nanolayers (MNLs) at inorganic interfaces offers promise for reaping unusual enhancements in fracture energy, thermal and electrical transport. Here, we reveal that multilayering MNL-bonded inorganic interfaces can result in viscoelastic damping bandgaps. Molecular dynamics simulations of Au/octanedithiol MNL/Au multilayers reveal high-damping-loss frequency bands at 33 ≤ ν ≤ 77 GHz and 278 ≤ ν ≤ 833 GHz separated by a low-loss bandgap 77 ≤ ν ≤ 278 GHz region. The viscoelastic bandgap scales with the Au/MNL interface bonding strength and density, and MNL coverage. These results and the analyses of interfacial vibrations indicate that the viscoelastic bandgap is an interface effect that cannot be explained by weighted averages of bulk responses. These findings prognosticate a variety of possibilities for accessing and tuning novel dynamic mechanical responses in materials systems and devices with significant inorganic–organic interface fractions for many applications, e.g., smart composites and sensors with self-healing/-destructing mechanical responses.

## Introduction

Molecular nanolayers (MNLs) have been extensively studied as *surfactants* for a variety of applications, such as, nanoparticle shaping, surface protection, tribology, lithography and electromechanical systems^1^. Several works over the last couple of decades have demonstrated that introducing MNLs at inorganic *interfaces* can lead to unexpected multifold enhancements in multiple properties^2–5^. Examples include MNL-induced fivefold fracture energy^2^ and fourfold contact thermal conductance^4^ increases at metal-oxide interfaces, and more than tenfold increases in electrical contact conductance at metal–semiconductor interfaces^5^. These remarkable enhancements correlate with strong bonding between the interfacial MNL and the inorganic layers.

Our recent work^3^ revealed interfacial MNL-induced toughening at select loading frequencies in polymer/metal/MNL/oxide structures, wherein the dynamic fracture energy under cyclic loading exceeds the static loading value. This unusual frequency response was traced to MNL-induced deformation in the proximal metal and the distal polymer layers. This led us to hypothesize that superposing such interface-derived responses in multilayers could lead to multiple frequency bands of viscoelasticity and/or fracture energy that could be tuned by manipulating the interfacial MNL chemistry and structure. Although frequency-dependent dielectrics properties^6^ and phononic responses and bandgaps^7^ are well known, such frequency-dependent mechanical features are new.

Here, we present molecular dynamics simulations showing that multilayers of MNL-bonded Au interfaces result in a low-damping viscoelastic bandgap between two high-damping frequency bands. These features are sensitive to the interface bonding strength and density, and are more than an order of magnitude different than values predicted by rules of mixtures. Thus, our results show that multilayering inorganic-MNL interfaces offer possibilities to access and tune novel frequency-dependent mechanical properties. These phenomena could be attractive for the design of smart sensors, actuators and composites^8^, e.g., with self-healing/-destructing mechanical responses^9^.

## Results and discussion

### Viscoelastic damping characterization

**Figure 1:**
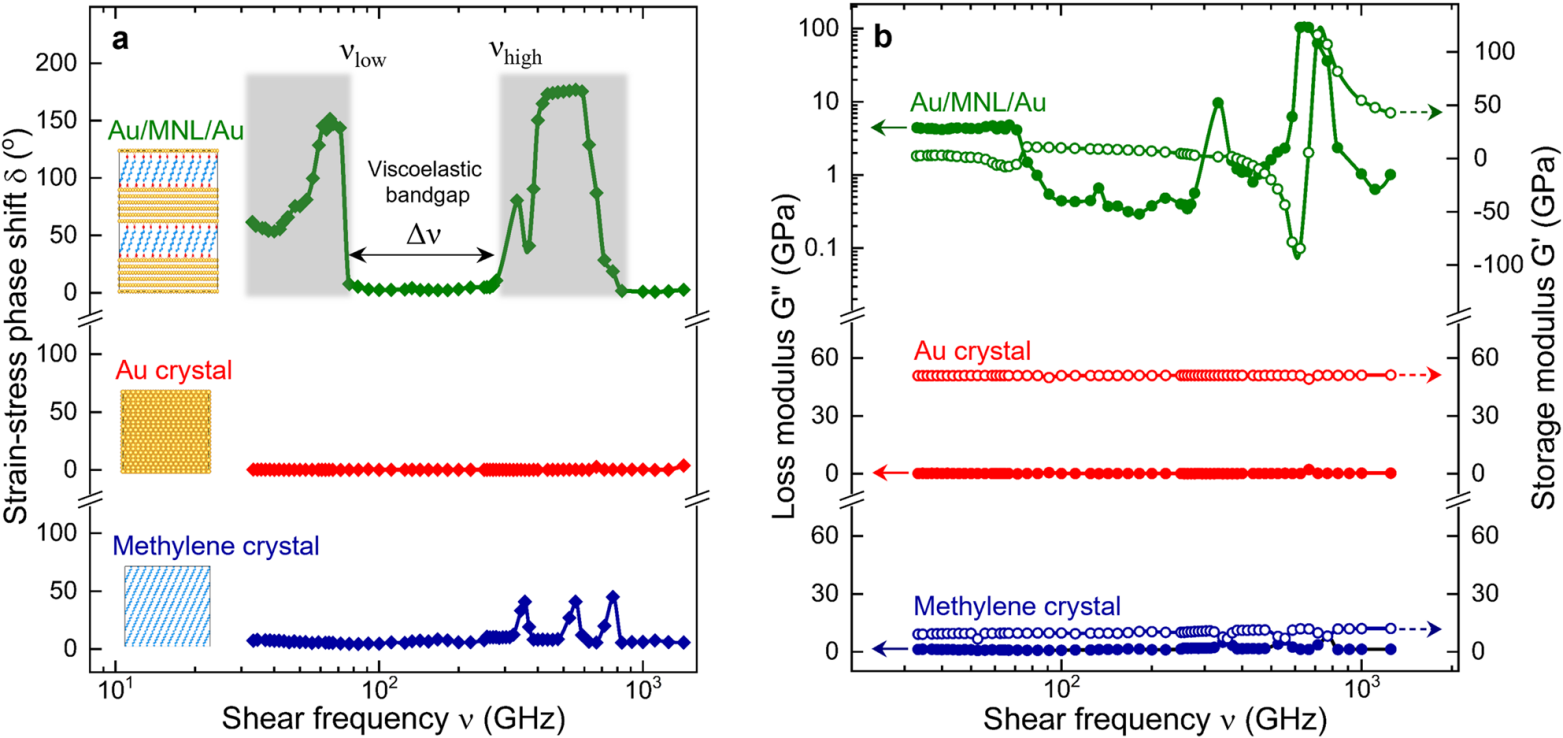
Viscoelastic bandgap in Au/octanedithiol MNL/Au multilayers. (**a**) Strain–stress phase shift in Au/octanedithiol MNL/Au multilayers, and the reference bulk crystals of Au and methylene chains as a function of frequency. The viscoelastic bandgap is given by Δν = ν_high_ − ν_low_ where ν_high_ and ν_low_ represent band edges. (**b**) Frequency-dependence of loss and storage moduli, G'' and G', respectively.

Molecular dynamics (MD) simulations (see “Methods”) were used to calculate the viscoelastic damping properties of Au/octanedithiol MNL/Au multilayer structure under GHz-frequency oscillatory shear strain. Loss and storage moduli were determined from the phase difference δ between shear strain and shear stress. Our results show frequency-dependent viscoelastic features (see Fig. 1) that are not seen in bulk crystals of either Au or methylene chains. Two strongly damping loss bands at 33 ≤ ν ≤ 77 GHz and 278 ≤ ν ≤ 833 GHz are separated by a low-damping bandgap Δν = ν_high_ − ν_low_ ~ 201 GHz, where ν_high_ ~ 278 GHz and ν_low_ ~ 77 GHz (see Fig. 1a).

Inside the viscoelastic bandgap, the low loss modulus G"_Au/octanedithiol MNL/Au_ ~ 0.5 GPa, and the storage modulus G'_Au/octanedithiol MNL/Au_ ~ 7.5 GPa; these lie within the range of values of bulk Au and methylene phases (Fig. 1b). Individual bulk crystals of Au exhibit elastic behavior G_Au_ ~ G'_Au_ ~ 51 GPa with minimal losses of G"_Au_ ≤ 0.25 GPa. Methylene chain crystals exhibit viscoelastic losses between 0.7 ≤ G"_MNL_ ≤ 8.2 GPa for the entire frequency range studied (Fig. 1b). The small peaks in the 40° ≤ δ ≤ 45° range, at ~ 357, ~ 556, and ~ 769 GHz (Fig. 1a) indicate temporal lags in the molecular assemblies in responding to applied stresses. The maximum loss G"_Au/octanedithiol MNL/Au_ ~ 104 GPa occurs at ν_max_ ~ 625 GHz, where G'_Au/octanedithiol MNL/Au_ exhibits frequency-dependent signatures typical of mechanical damping processes (Fig. 1b).

The damping energy losses were, however, not only unlike that expected for bulk Au or methylene chains, but also entirely different from estimates obtained using series and parallel rules of mixtures (ROM)^10^. The parallel model defines the lower bound shear modulus magnitude G_LowerROM_ ~ 15 to 20 GPa for 33–1429 GHz, while the series model yields the upper bound G_UpperROM_ ~ 30 to 33 GPa. Our simulations show that G_Au/octanedithiol MNL/Au_ is either two-to-three-fold lower than G_LowerROM_, or four-to-nine-fold higher than G_UpperROM_. This result indicates that damping in the Au/octanedithiol MNL/Au multilayer is predominantly governed by interface effects, and cannot be explained by weighted averages of bulk responses.

### Interfacial bond density

**Figure 2:**
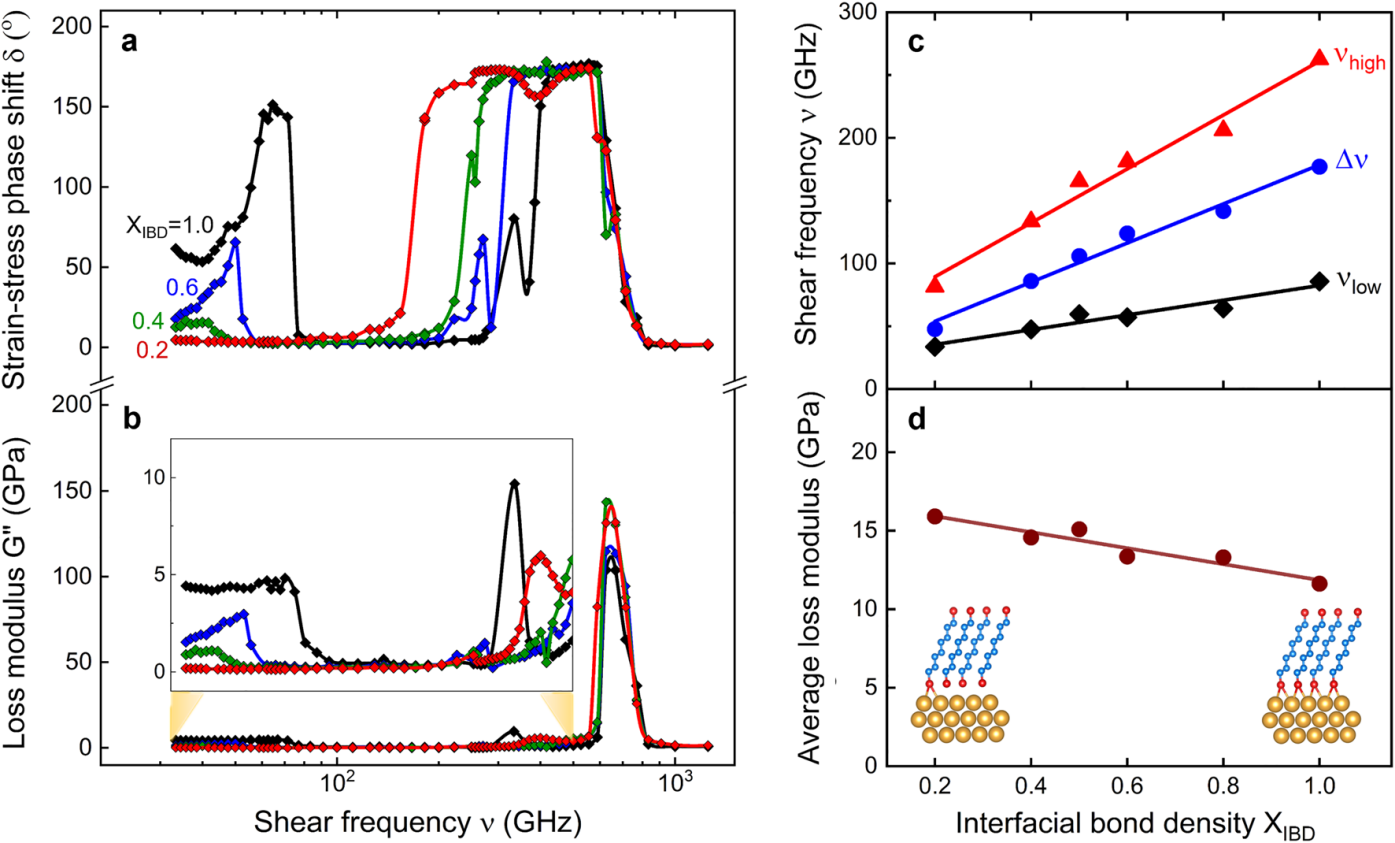
Viscoelastic damping characteristics in Au/octanedithiol MNL/Au multilayers during oscillatory shear for different interfacial Au–S bond densities, X_IBD_. **(a**) Strain–stress phase shift δ and (**b**) loss modulus G'', as a function of shear frequency. (**c**) Viscoelastic damping gap Δν, and the band edges ν_high_ and ν_low_, and (**d**) frequency-averaged loss modulus as function of X_IBD_.

The low-damping viscoelastic bandgap magnitude Δν and the band edge positions ν_low_ and ν_high_ are sensitive to the Au–S interfacial bond density X_IBD_ (Fig. 2a). Increasing X_IBD_ correlates with linear increases in the bandgap Δν = ν_low_ − ν_high_ and the high-damping band edge frequencies (see Fig. 2b,c). Furthermore, increasing X_IBD_ correlates with an increase in the low-frequency band damping magnitude and a decrease in the high-frequency band width. A consequence of these behaviors is that the frequency-averaged loss modulus G'' decreases linearly with X_IBD_ from ~ 16 GPa to ~ 11.6 GPa for 0.2 ≤ X_IBD_ ≤ 1 (see Fig. 2d). Essentially, identical results (not shown) are obtained when we vary the bond strength connoted by X_IBS_. In our simulations, varying bond strength does not involve bond elimination, as is the case when altering bond density.

### Interfacial molecular coverage

**Figure 3:**
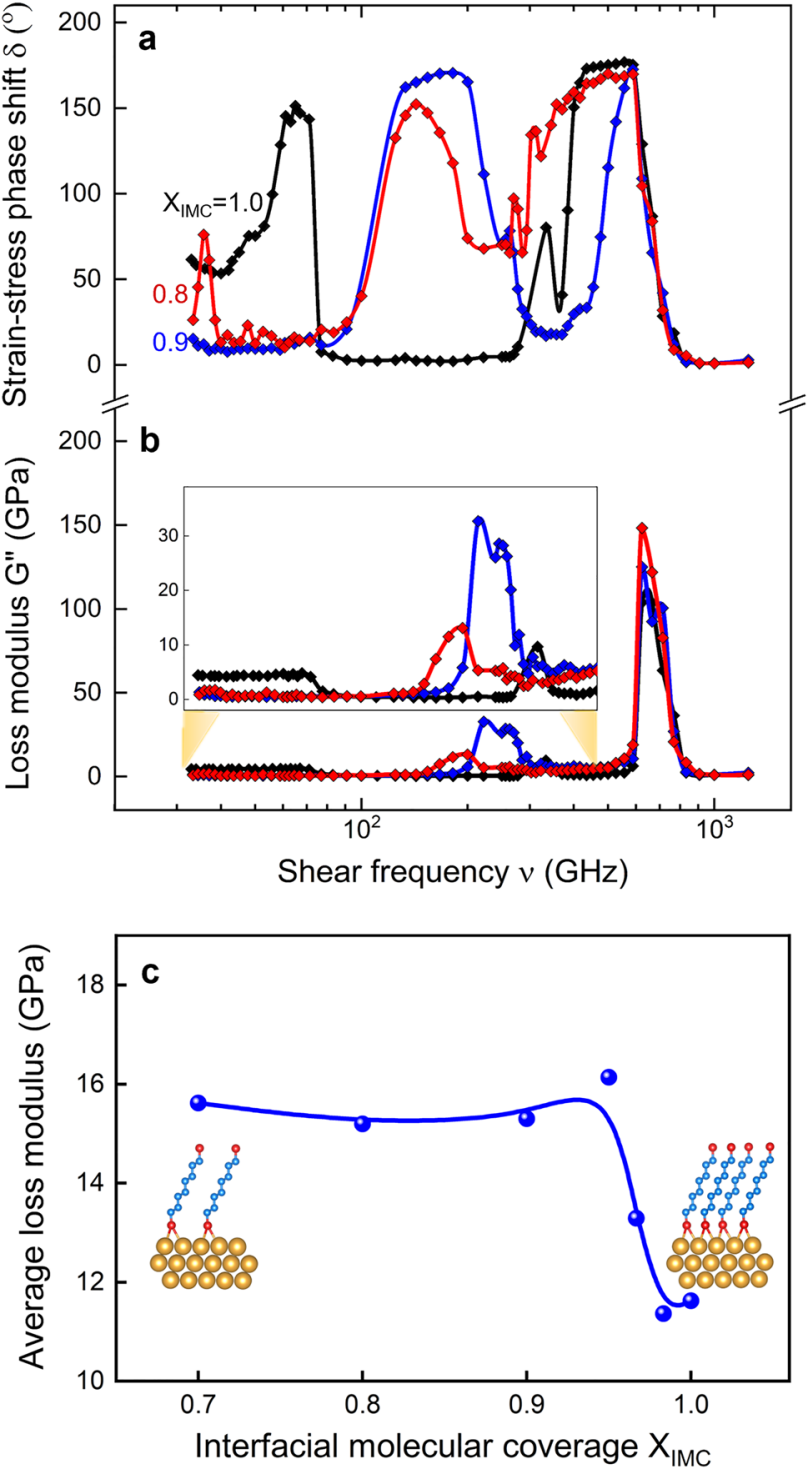
Viscoelastic damping characteristics in Au/octanedithiol MNL/Au multilayers for different interfacial molecular coverages X_IMC_. **(a**) Strain–stress phase shift δ and (**b**) loss modulus G'' as a function of shear frequency, (**c**) frequency-averaged G'' as function of X_IMC_.

Decreasing the interfacial molecular coverage X_IMC_ by removing entire molecules resulted in a greater effect (see Fig. 3) on the viscoelastic bandgap than that obtained by deactivating interfacial bonds (i.e., by decreasing X_IBS_ and X_IBD_). For example, the viscoelastic bandgap completely disappears upon decreasing X_IMC_ by ~ 10%. This result is consistent with^11,12^ the higher average energy dissipation per unit coverage expected upon decreasing molecular coverage. These findings indicate that both the molecular packing, molecular order, and interfacial bond strength strongly influence viscoelastic bandgap magnitude and position. These results point to new vistas for creating a rich variety of materials with tailored frequency-dependent viscoelastic properties.

### Vibrational density of states

**Figure 4:**
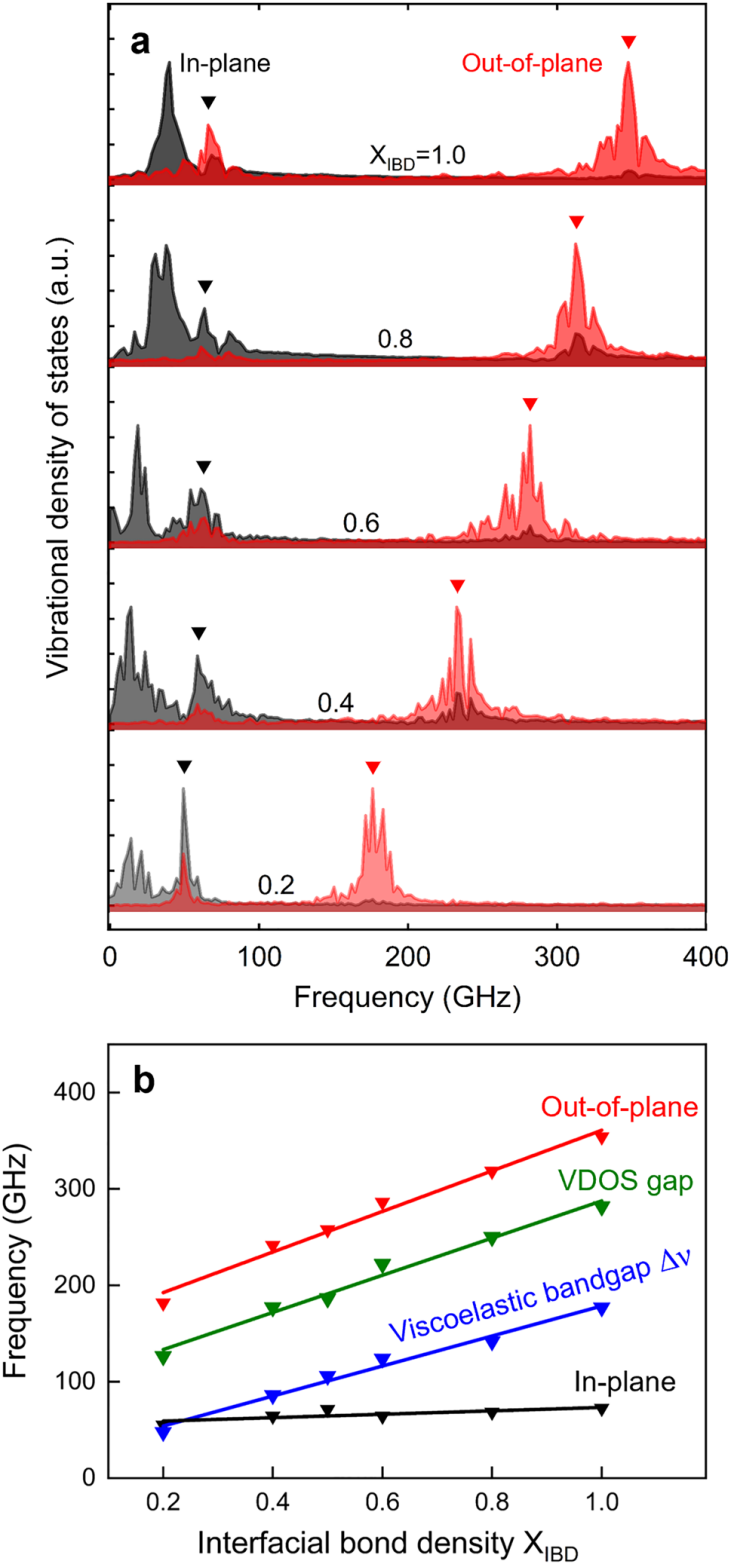
Vibrational density of states (VDOS) of center of mass of gold. **(a**) VDOS spectra from gold in the Au/octanedithiol MNL/Au multilayers for different bond densities, X_IBD_, indicating in-plane and out-of-plane modes. (**b**) In-plane and out-of-plane frequency as a function of Au–S bond density. The VDOS gap is the difference between out-of-plane and in-plane frequency peaks, and has the same trend as the viscoelastic damping gap replotted from Fig. 2c.

Calculations of the vibrational density of states (VDOS) of the Au-block center-of-mass relative to the Au/MNL interface reveals that the viscoelastic deformation to be accommodated exclusively by the interface. The out-of-plane interfacial vibration peak position shifts monotonically to higher frequencies from ~ 182 to ~ 354 GHz, with increasing interfacial strength denoted by X_IBD_ (see Fig. 4a,b). The two in-plane low-frequency modes in the 15–75 GHz range also change with X_IBD_ but to a lesser extent. No VDOS features are observable within the low-damping viscoelastic bandgap. Phonon spectra computed by considering the motion of all atoms in the system reveal no phononic bandgaps in the GHz range (not shown), indicating that the viscoelastic bandgap is not related to Au or MNL phonon modes, but arises from *global* damping losses in the *entire* multilayered Au/octanedithiol MNL/Au structure.

The gap between in- and out-of-plane interfacial mode peaks (Fig. 4a) in vibrational density of states (VDOS) shows similar sensitivity to the bond density (Fig. 4b) as the viscoelastic gap plotted in Fig. 2c. The fact they there are not quantitively the same is expected as the VDOS is evaluated for a specific subset of interfacial motion associated with relative displacement of the whole Au and MNL slabs. Strong correlations between the viscoelastic gap and the vibrational gap provide another and powerful evidence for interfaces being centrally responsible for the remarkable mechanical responses of the organic–inorganic nanolayers revealed in this work.

### Spatial power loss

**Figure 5:**
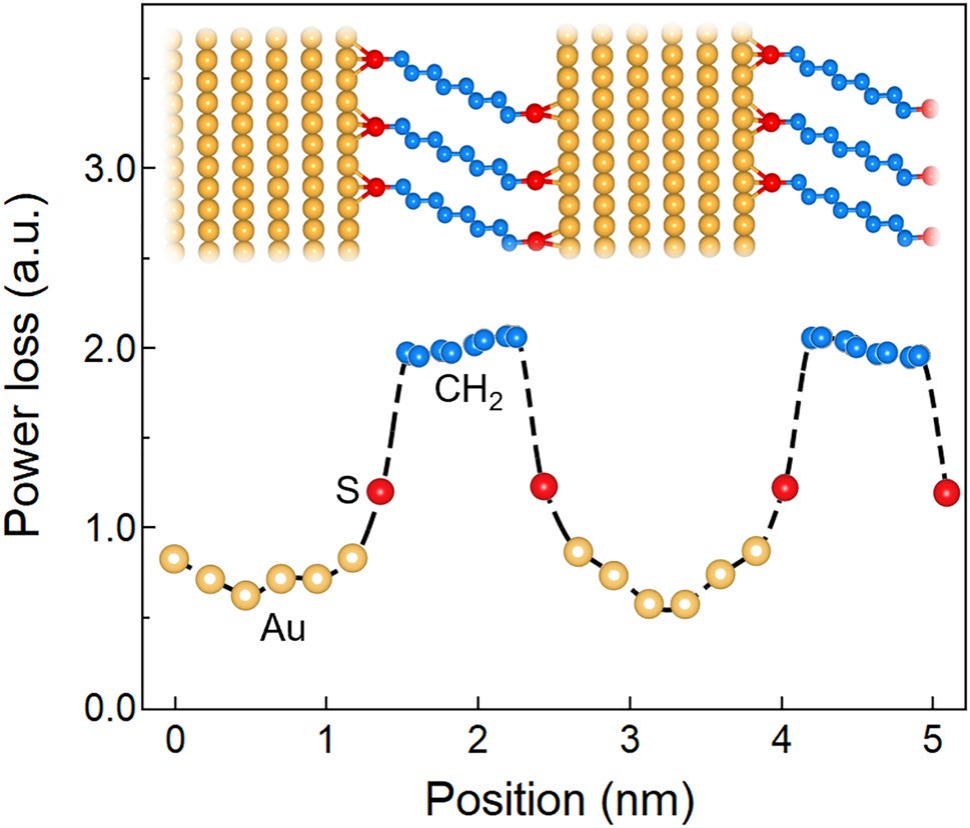
Spatial power loss in Au/octanedithiol MNL/Au multilayers. The energy loss rate was determined for oscillatory shear at 625 GHz corresponding to the maximum loss modulus G''.

In order to obtain insights into interface-induced damping, we spatially mapped energy dissipation using a Berendsen thermostat and averaging the power loss rate over atomic planes across the Au/octanedithiol MNL/Au structures. Our results (see Fig. 5) indicate 2 to 3.5-fold higher power losses in the MNLs than in the Au nanolayers, with a large interfacial power loss gradient. Higher loss in the MNL is consistent with the availability of multiple energy dissipation modes in MNLs, e.g., chain kinking, bending and rotation^13^. No such losses are observed in methylene crystal ensembles, indicating that energy loss occurs primarily in the organic MNL, but only when strongly bonded to the inorganic nanolayers.

## Conclusion

In conclusion, our study has unearthed the existence of viscoelastic damping bandgaps in Au/octanedithiol MNL/Au multilayers subject to cyclic shear. The bandgap features are sensitive to, and tunable with, interfacial bond strength and density, and molecular coverage. The viscoelastic bandgap scales with the Au/MNL interfacial bonding strength and density, and MNL coverage. Our results provide compelling evidence that this behavior is an intrinsically interfacial effect that cannot be explained in terms of weighted averages of bulk responses. These findings presage a variety of possibilities for accessing and tuning novel dynamic mechanical responses in materials systems and devices with significant inorganic–organic interface fractions for applications.

## Methods

### Model creation

We created a unit-cell consisting of four octanedithiol molecules with the sulfur head group adsorbed on the face centered cubic hollow sites of ($\sqrt{3}\times \sqrt{3}$) R30° surface of Au(111) using methods detailed elsewhere^15,16^. The unit-cell was repeated to 6 $\times$ 5 $\times$ 2 along x-, y-, and z-axes using the Moltemplate builder^14^ to create a 5.18 $\times$ 4.98 $\times$ 5.32 nm^3^ cell with Au/octanedithiol MNL/Au multilayers (see Fig. 1a). Each multilayer consists of ~ 1.2-nm-thick single-crystal Au(111) nanolayers bonded with ~ 1-nm-thick octanedithiol MNLs. The methylene groups in the octanedithiol backbone were treated as superatoms, wherein the effects of individual hydrogen atoms were not considered. We used force-field parameters for describing pairwise bonding, non-bonding^15^, and dihedral^17^ interactions.

### MD protocol

We carried out the molecular dynamics (MD) calculations using the LAMMPS package^18^. Firstly, we equilibrated the model structures for 400 picoseconds at 300 K at zero pressure using an isothermal-isobaric ensemble (NPT) and applied GHz-frequency shear strain γ = γ_0_sin2πνt on a canonical ensemble (NVT) for 1000 cycles using a method described elsewhere^8,19^. The oscillatory shear resulted in a strain amplitude γ_0_ = 1% for 33 ≤ ν ≤ 1429 GHz. Our simulations used a 2-fs integration time step and a Berendsen thermostat with a 5 ps damping timescale, with periodic boundary conditions applied in all the directions.

Series and parallel rules of mixtures were used for computing the upper and lower bound shear modulii of the Au/octanedithiol MNL/Au multilayers, i.e., G_UpperROM_ = V_Au_G_Au_ + V_MNL_G_MNL_ and G_LowerROM_ = G_Au_G_MNL_/(V_Au_G_Au_ + V_MNL_G_MNL_) respectively.

We obtained the vibrational density of states (VDOS) of the relative motion of Au nanolayers about the Au/MNL interfaces by Fourier transforming the velocity autocorrelation function determined from the Au center of mass velocities monitored for 500 pico-second during equilibrium in our microcanonical ensemble (NVE) simulations.

## Data Availability

All the data generated or analyzed during the current study are available from the corresponding author upon reasonable request.
